# Species Discrimination among Three Kinds of Puffer Fish Using an Electronic Nose Combined with Olfactory Sensory Evaluation

**DOI:** 10.3390/s120912562

**Published:** 2012-09-13

**Authors:** Meixiu Zhang, Xichang Wang, Yuan Liu, Xinglian Xu, Guanghong Zhou

**Affiliations:** 1 College of Food Science and Technology, Shanghai Engineering Research Center of Aquatic-Product Processing & Preservation, Shanghai Ocean University, Shanghai 201306, China; E-Mails: mxzhang68023762@yahoo.com.cn (M.Z.); xcwang@shou.edu.cn (X.W.); 2 National Center of Meat Quality and Safety Control, Nanjing Agricultural University, Nanjing 210095, China; E-Mails: xlxu@njau.edu.cn (X.X.); ghzhou@njau.edu.cn (G.Z.)

**Keywords:** puffer fish, electronic nose, olfactory sensory evaluation, principal component analysis (PCA), discriminant factorial analysis (DFA)

## Abstract

Species discrimination among three kinds of puffer fish, *Takifugu obscurus*, *Takifugu flavidus* and *Takifugu rubripes*, was conducted using an electronic nose combined with olfactory sensory evaluation. All data were treated by multivariate data processing based on principal component analysis (PCA) and discriminant factor analysis (DFA). The results showed the discriminant model by PCA method and DFA method. Using PCA and DFA, it was shown that the electronic nose was able to reasonably distinguish between each of the eleven puffer fish groups, with a discrimination index of 85. The olfactory sensory evaluation was undertaken in accordance to Sensory analysis—Methodology—Initiation and training of assessors in the detection and recognition of odors (BS ISO 5496-2006), and the results showed that the evaluation was able to identify puffer fish samples according to their species, geographical origin and age. Results from this analysis demonstrate that the E-nose can be used to complement the discrimination of odors by sensory evaluation from the three species of puffer fish studied here.

## 1. Introduction

There are more than 100 species of puffer fish worldwide and about 22 species genus *Takifugu* are distributed along the coastal waters of China [1,2]. Three common edible species of puffer fish, *Takifugu obscurus*, *Takifugu flavidus* and *Takifugu rubripes*, are cultured in various locations in the Yangtze River, the East Sea, the Yellow Sea, the South Sea and the Bohai Sea of China. These cultured puffer fish, which are very popular among consumers, not only have a high growth rate but yield non-toxic meat, which still retains a delicious taste. They are regarded as a high quality fish because of their high protein content and special flavor. On the one hand, since “cross-breeding” technology is commonly applied to some species of puffer fish, resulting in hybrids or some intermediate varieties could not be recognized by morphological characteristics [3]. On the other hand, with the improvement of living conditions, the consumers have the high expectation for the quality of cultured puffer fishes. However, it is quite difficult to classify the species of the puffer fish and describe the quality of puffer fish. Few studies are reported up to now on the application of electronic nose technology to identify the puffer fish.

Odor is generally understood to be the overall experience from nasal stimulation and is principally derived from the human senses of smell (olfaction) [4]. Sensory and instrumental techniques, using panelists and/or gas chromatography (GC), are commonly used to determine the odor of food products [5,6]. However, these measurements are often time-consuming, expensive and sometimes are without any objective value [7]. Also, a human sensory panel may be limited in its ability to detect volatile compounds that are non toxic or not obnoxious.

Puffer fish of different species, geographical origin and age contain different volatile compounds that contribute to their specific odors which are contributed to by the meat of the puffer fish. The electronic nose (E-nose) has been widely used for recognizing odors by way of specific sensors [8,9]. The E-nose has been shown to be effective for determining the “volatile fingerprint” of a product based on (for example) levels of CO, NO/NO_2_, CH_4_, H_2_S, *etc.* as well as volatiles, and is capable of displaying excellent discrimination. The sensor arrays of the E-nose provide an output pattern that represents the combined outputs from the components [10]. The output pattern is given by the selectivity of the various sensors [11]. Although the specificity of each sensor may be low, the combination of a sensor array, each with a different selectivity pattern, provides a large amount of information, thus allowing detection of a very large number of odors [12]. E-noses have been proven to be a useful instrumental technique for the food and drinks industry for product discrimination, classification, quality evaluation and control [13,14]. The main advantages of the E-nose are that it is rapid and objective and provides overall information. To prevent adulteration and to maintain the safety aspects of species identification and classification of puffer fish for human consumption, it is necessary to develop rapid, low cost, easy-to-handle and objective methods for their identification.

In this paper, an innovative, rapid and objective analytical technique consisting of the E-nose coupled with an olfactory sensory evaluation was used to differentiate the species and objective sensorial evaluation of three species of puffer fish. For this purpose, E-nose signals were analyzed by principal component analysis (PCA) and discriminant factorial analysis (DFA), and the data of the olfactory sensory evaluation was treated by the SPSS 17.0 software.

## 2. Experimental Section

### 2.1. Materials and Sample Preparation

Cultured puffer fish including *Takifugu obscurus*, *Takifugu flavidus* and *Takifugu rubripes* were chosen for this study. Fifty-two puffer fishes were purchased from Nengzheng Group Co., Ltd. (Shanghai, China), Wusi farm (Shanghai, China), Zhongyang Group Co., Ltd. (Nantong, Jiangsu province, China) and Qinhuangdao (Hebei province, China) (shown in Table 1). All puffer fish were transported alive to the laboratory, and killed instantly. Body meat was packed in aluminum foil, then sealed in plastic bags under vacuum and stored at −80 °C until required for analysis. All puffer fish were grouped into eleven categories according to species, geographical origins and age.

Puffer fish meat packed in sealed plastic bags was thawed by flowing tap water for 30 min and then homogenized using a meat grinder (A11, IKA, Germany). For the olfactory sensory evaluation, 50 g of each homogenized meat sample was added to 400 mL de-ionized water and then kept at 60 °C for 20 min, followed by 100 °C for 40 min until the odor could be detected. Following these treatments, the aqueous extract of the puffer fish was used for sensory evaluation of odor.

### 2.2. The E-Nose Measurements

The E-nose used for this study was an E-nose αFOX 4000 (Alpha MOS, France). It is an odor and volatile organic compounds (VOC) analyzer. The E-nose consists of a headspace autosampler HS100 with numerous options, 18 metal oxide sensors with different selectivity patterns, a signal collecting unit and pattern recognition software applied via a computer.

There were 11 groups of puffer fish samples and each group was prepared three times at different times. For each prepared sample at one time, there were seven duplicates. Only the last three duplicates were averaged into one point, totaling three points for each sample in the PCA and DFA plot. The sample order for the PCA and DFA plots was alphabetical. The E-nose showed good stability for each of the three replicates. For E-nose analysis, each sample, 2.06 ± 0.03 g of homogenized meat was placed in an 18 mm precision thread vial (10 mL) equipped with a magnetic screw-thread cap (CNW Technologies GmbH, Düsseldorf, Germany). A number of preliminary tests were made in order to determine the optimum conditions that were acceptable for all of the samples using PCA analysis (Table 2).

### 2.3. Olfactory Sensory Evaluation

Olfactory sensory evaluation was conducted essentially as described by the Sensory analysis—Methodology—Initiation and training of assessors in the detection and recognition of odors (BS ISO 5496-2006) using the direct smelling method. The olfactory sensory evaluation panelists consisted of five males and six females (age 19–23) who had previously been trained to recognize the odor attributes, and were known for their accurate sensory evaluation abilities. Subsequent analyses of the puffer fish samples were performed in triplicate on different days.

For each sample, 5 mL puffer fish aqueous extract was placed in a 15 mL brown glass flask equipped with a non-lubricated ground-glass stopper. These brown glass flasks have sufficient capacity to hold the products to be tested and to leave sufficient head space to permit equilibrium of the vapor pressure. The samples were prepared at least 30 min before the test to allow time for the vapor pressure to reach equilibrium at ambient temperature. The panelists were asked to open the flasks one by one, with their mouths closed and to sniff the vapor phase of the puffer fish aqueous extract. The panelists were also asked to smell the flasks in short sniffs or deep breaths at suitable time intervals in a similar way. Once a decision had been made on identity, the flasks were stoppered. Following sensory detection, the eleven panelists were asked to describe the odor and provide a score for each sample. Eight odors attributes, including fish meat-like, nut-like, chicken meat-like, fishy, forest damp soil-like, crab meat-like, fatty and butter-like smell were developed in accordance to the preliminary olfactory sensory evaluation by panelists. The following scores were used to rank the intensity of these attributes: very strong-50, strong-40, fairly strong-30, weak-20, very weak-10.

### 2.4. Statistical Analysis

For the E-nose data, principal component analysis (PCA) and an unsupervised method, were used for data visualization and the detection of groups in the data structure. PCA was used to remove the redundancy of variables and to give a representative map of the different areas. The discrimination index was used to indicate the extent of discrimination between samples on the two-dimensional PCA surface. In addition, it was possible to classify the samples without any prior information on the samples [9]. Supervised analysis, such as DFA, was used to make reliable recognitions for unknown samples. Conversely, DFA requires prior knowledge regarding the samples. The models obtained from DFA analysis were validated using a modified cross-validation (leave-one-out) method. The data were processed by a statistical software program (Alpha Soft Version 12.3).

For the sensory data, means were calculated and statistically tested using analysis of variance (ANOVA). If a statistical difference existed at *P* < 0.05, Duncan's multiple range tests were used to identify the statistical separation among the means. The statistical analysis was performed with the software SPSS 17.0.

## 3. Results and Discussion

### 3.1. Odor Evaluation by E-Nose with PCA and DFA

E-nose data contains overlapped information. This problem can be solved by a multivariate data analysis such as PCA which is an unsupervised pattern recognition [15]. PCA are used to remove the redundancy of variables and to give a representative map of the different olfactive areas. It could transform the original measured variables into new uncorrelated variables called principal components, and allows data visualization retaining as much as possible the information present by the reduction of the data dimensionality [16]. Each principal component is a linear combination of the original measured variables. The first principal component (PC1) accounts for the maximum of the total variance, the second (PC2) is uncorrelated with the first and accounts for the maximum of the residual variance, and so on, until the total variance is accounted for. For practical reasons, it is sufficient to retain only those components that account for a large percentage of the total variance. The values that represent the samples in the space defined by the principal components are the component scores. The discrimination index indicates the extent of discrimination between samples in the two-dimensional PCA surface. In addition, the samples can be classified without prior information on the samples when using PCA. Conversely, DFA require prior knowledge about the samples. DFA was used to determine whether it is possible to separate two or more individual groups, given measurements for these individuals from several variables [17]. PCA treats each replicate samples as individual data, but the DFA assume replicate samples are clustered.

Initially, the discriminative ability of the E-nose system was applied to distinguish the odors of all the puffer fish. Eleven puffer fish samples A1-WS, A2-WS, A2-NZ, A2-ZY, J1-WS, J2-WS, J1-QH, J2-QH, J2-NZ, H1-QH and H2-QH were examined and analyzed by E-nose with PCA and DFA as the analysis method. PCA was performed on the initial instrumental data to explore the connection and relationship of each data set.

**Figure 1.**
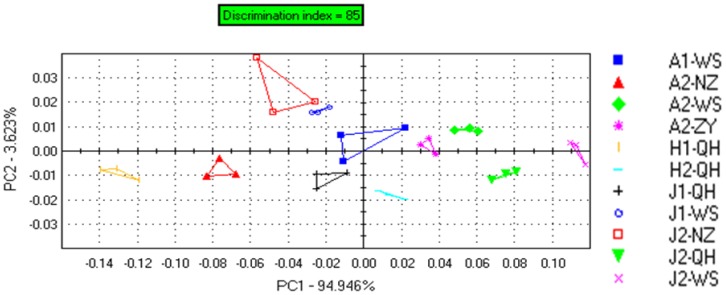
PCA plot for the E-nose results for each of the eleven groups of puffer fish.

The PCA score plot in Figure 1 shows that the E-nose effectively discriminates each of the puffer fish groups. The first two principal components PC1 (94.946%) and PC2 (3.623%) explain 98.569% of total system variance. According to the statistical model, a successful discrimination model should have an index between 80 and 100 [18]. The discrimination index was 85 (maximum 100), indicating that a certain degree of discrimination was achieved. PCA shows that each of the eleven puffer fish samples groups was discriminated from the each other. Puffer fish samples from the same origin were closely located on the PCA plot, forming a group. At the same time, different samples which are from the same group are not completely overlapping each other, indicating the greater sensitivity of the E-nose. The same varieties of the same age, but from different origins, do not group together in the PCA plot. These reasons that came to such phenomena are made clear by difference of the geographical origins. The climate and cultured waters in south China are very different from north China and the distance between the geographical origins will influence the odors of the puffer fish.

**Figure 2.**
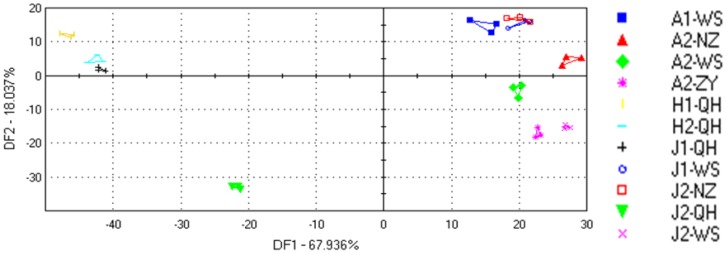
DFA plot for the E-nose results for each of the eleven groups of puffer fish.

DFA is a procedure that maximizes the differences between groups, and the capability of DFA to discriminate between the groups of puffer fish was also good (Figure 2). The puffer fish sample groups of A1-WS, A2-WS, A2-NZ, A2-ZY, J2-WS, J1-QH, J2-QH, H1-QH and H2-QH could be separated very well and samples identification recognition percentage using DFA among these samples was higher than 90%.

The ability to distinguish between different samples depends on the distance between the centers of the clusters. The recognition percentage was calculated to indicate a relatively accurate identification of the various unknown samples. Moreover, the use of DFA had an improving effect among these samples as compared with the PCA, as has been found by Huang [19]. However, puffer fish group J1-WS had a recognition percentage of less than 90% when compared with J2-NZ, thus samples J1-WS and J2-NZ could not be distinguished by the E-nose when using the DFA recognition pattern. This may indicate that the samples J1-WS and J2-NZ contain similar volatile compounds.

### 3.2. Olfactory Sensory Evaluation

**Figure 3.**
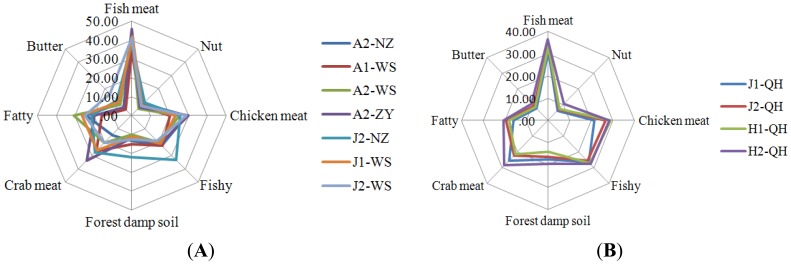
Radar plots for sensory scores of cultured puffer fish.

Using ANOVA and Duncan's multiple range tests, the statistical separation analysis for the attributes of the puffer fish samples is shown in Table 3. Based on this table, two spider plots were created to provide a graphic representation of the odor profiles. Thus, the differences and similarities of the puffer fish samples are readily identified in Figure 3. The sensory data contained information related to the varieties, geographical origins and age.

For the sensory evaluation of puffer fish, there were statistically significant differences (*P* < 0.05) for the attributes, fish meat-like, fishy, crab meat-like, and fatty smell, used to describe the odor of the puffer fish aqueous extracts. However, the attributes, chicken meat-like, forest damp soil-like, nut-like and butter-like smell, were non-significantly different. These four attributes were unable to be separated in the puffer fish samples. Moreover, the sensory scores of these four attributes were lower than for the other four attributes. Low concentrations of odor compounds may have been the reason for the inability to classify puffer fish samples based on these four attributes.

For the olfactory sensory evaluation results of the *Takifugu obscurus*, the odors of A1-WS and A2-WS were different from the attributes of fish meat-like and fatty smell. A2-WS and A2-ZY were differentiated by their odor attribute of fatty smell, and A2-NZ and A2-ZY were distinguished by their odor attribute of crab meat-like smell. There was no significant difference between A2-WS and A2-NZ among any of the eight odor attributes.

For the olfactory sensory evaluation of *Takifugu flavidus*, J1-WS and J1-QH were separated by the odor attributes of fish meat-like and fatty smell. The odors of J2-WS and J2-NZ were different from fishy smell. There were no significant differences between the eight odors attributes for J1-WS and J2-WS, J1-QH and J2-QH, J2-WS and J2-QH, and for J2-QH and J2-NZ.

For the olfactory sensory evaluation of *Takifugu flavidus*, the odors of H1-QH and H2-QH were not different based on the eight odor attributes.

The olfactory sensory evaluation of odors revealed that J1-WS and J2-WS, J1-QH and J2-QH, H1-QH and H2-QH, A2-WS and A2-NZ, J2-WS and J2-QH, J2-QH and J2-NZ were all very similar and the samples could not be discriminated by the panelists. However, A1-WS and A2-WS, A2-WS and A2-ZY, A2-NZ and A2-ZY, J1-WS and J1-QH, J2-WS and J2-NZ could be distinguished by their odor attributes. On the one hand, these results demonstrated that the odors of *Takifugu obscurus* which were farmed in fresh water were influenced by age, but the odors of *Takifugu flavidus* and *Takifugu rubripes* which were cultivated in brackish water or salt water were not affected by age. Whilst, geographical origin had some influence on the odors of the *Takifugu obscurus* and *Takifugu flavidus*, the influence on the olfactory sensory evaluation of A2-WS and A2-NZ, J2-WS and J2-QH, J2-QH and J2-NZ could not be demonstrated.

When comparing the E-nose determinations with olfactory sensory evaluation, all of the puffer fish samples could be discriminated by the PCA. However J1-WS and J2-NZ were unable to be separated (*P* < 0.05) by the E-nose when using the DFA for any of the eight attributes (Table 3). Most importantly, samples A2-WS and A2-NZ, J1-WS and J2-WS, J1-QH and J2-QH, J2-WS and J2-QH, J2-QH and J2-NZ, H1-QH and H2-QH were separated by the E-nose using the PCA, although they were not discriminated by olfactory sensory evaluation. These findings indicate that the discrimination ability of the instrument is greater than that of the human nose. Results from these analyses demonstrate that the E-nose represents a valuable addition to complement sensory evaluation for the discrimination of odors derived from these three types of puffer fish.

## 4. Conclusions

A FOX 4000 E-nose using recognition patterns with PCA and DFA, combined with an olfactory sensory evaluation, has been applied for the classification of puffer fish derived from various species, different geographical locations and age. It has been found that the E-nose, coupled with olfactory sensory evaluation, is capable of identifying each of the puffer fish groups, and that the E-nose could be used to complement olfactory sensory evaluation. Although the E-nose could discriminate between the different puffer fish groups, it was unable to indicate which factors resulted in the different odors of different fish groups. Other measurements such as GC-MS will be used in further research. The approach used in these experiments with the E-nose could be applied to discriminate other fish, particularly for wild poisonous fish.

## Figures and Tables

**Table 1.** Background information on the 11 puffer fish groups used.

**Species**	**Sample codes**	**Geographical origin**	**Age**	**Number**	**Body weight Mean ± SD (g)**
*Takifugu obscurus*	A1-WS	Wusi farm, Shanghai	1	6	94.08 ± 9.40
	A2-WS	Wusi farm, Shanghai	2	3	267.00 ± 8.54
	A2-NZ	Nengzheng, Shanghai	2	3	255.52 ± 4.82
	A2-ZY	Zhongyang, Jiangsu province	2	5	299.36 ± 9.16

*Takifugu flavidus*	J1-WS	Wusi farm, Shanghai	1	7	74.50 ± 13.74
	J2-WS	Wusi farm, Shanghai	2	3	275.20 ± 65.55
	J1-QH	Qinhuangdao, Hebei province	2	7	146.98 ± 28.39
	J2-QH	Qinhuangdao, Hebei province	2	4	225.19 ± 47.01
	J2-NZ	Nengzheng, Shanghai	2	3	238.00 ± 3.00

*Takifugu rubripes*	H1-QH	Qinhuangdao, Hebei province	1	7	173.99 ± 44.31
	H2-QH	Qinhuangdao, Hebei province	2	4	489.60 ± 43.19

**Table 2.** Analytical conditions with the αFOX 4000 system.

Quantity of sample in the vial	2.06 ± 0.03 g
Total volume of the vial	10 mL
Headspace generation time	600 s
Headspace generation temperature	50 °C
Agitation speed	500 rpm
Acquisition duration	120 s
Acquisition period	1.0 s
The time between injections	600 s
Flow speed	150 mL/min
Injected volume	1500 μL
Injection speed	1500 μL/s
Syringe temperature	60 °C

**Table 3.** Means for olfactory sensory evaluation of puffer fish samples.

**Varieties**	**Sample codes**	**Fish meat-like**	**Fishy**	**Crab meat-like**	**Fatty**	**Nut-like**	**Chicken meat-like**	**Forest damp soil-like**	**Butter-like**
*Takifugu obscurus*	A1-WS	34.17^ab^	22.64^ab^	26.67^ab^	15.83^a^	6.67	20.14	15.28	4.58
	A2-WS	44.31^c^	19.86^a^	20.42^ab^	30.56^e^	5.28	25.28	10.28	8.33
	A2-NZ	36.67^abc^	22.22^ab^	14.44^a^	24.17^bcde^	8.89	28.61	13.19	9.44
	A2-ZY	45.83^c^	21.25^ab^	33.89^b^	20.14^abcd^	6.11	29.86	12.08	6.11

*Takifugu flavidus*	J1-WS	41.67^bc^	21.39^ab^	26.25^ab^	26.53^de^	7.22	22.92	11.39	10.00
	J2-WS	40.97^bc^	19.00^a^	20.42^ab^	24.86^cde^	7.08	28.89	12.78	16.11
	J1-QH	31.25^a^	26.53^ab^	25.69^ab^	16.11^ab^	5.97	21.25	17.50	7.64
	J2-QH	34.03^ab^	25.69^ab^	22.25^ab^	19.56^abcd^	6.94	26.39	16.42	9.58
	J2-NZ	38.33^abc^	33.33^b^	27.64^ab^	21.25^abcd^	9.44	25.56	22.22	10.97

*Takifugu rubripes*	H1-QH	33.47^ab^	27.36^ab^	21.53^ab^	18.06^abc^	7.50	28.75	14.03	8.47
	H2-QH	36.53^abc^	27.78^ab^	28.61^b^	20.56^abcd^	10.42	28.33	19.72	10.69

Note: Data in the same column with different letters are significantly different (*P* < 0.05, n = 3, Duncan's method).
